# A Typology of Buyers Grounded in Psychological Risk Factors for Compulsive Buying (Impulsivity, Self-Esteem, and Buying Motives): Latent Class Analysis Approach ina Community Sample

**DOI:** 10.3389/fpsyt.2020.00277

**Published:** 2020-04-21

**Authors:** Gaëlle Challet-Bouju, Julie Mariez, Bastien Perrot, Marie Grall-Bronnec, Emeline Chauchard

**Affiliations:** ^1^ CHU Nantes, Addictology and Psychiatry Department, Nantes, France; ^2^ Université de Nantes, Université de Tours, INSERM, SPHERE U1246 “methodS in Patient-centered outcomes and HEalth ResEarch”, Nantes, France; ^3^ Université de Nantes, Laboratoire de Psychologie des Pays de la Loire, Nantes, France

**Keywords:** compulsive buying, behavioral addictions, latent class cluster analysis, motivation, impulsivity, negative reinforcement

## Abstract

Our objective was to identify meaningful subgroups of buyers based on psychological risk factors for compulsive buying. A community sample of 242 adult women fulfilled an online survey exploring buying habits and motives, impulsivity, self-esteem, and severity of compulsive buying. A latent class cluster analysis was performed. A nonproblematic cluster (28%) was characterized by low levels of impulsivity and buying motives. An intermediary cluster (51%) was characterized by higher levels of positive and negative reinforcement-related buying motives. Both clusters were characterized by a low frequency of compulsive buying (2 and 8%, respectively), but the severity of compulsive buying was higher for the intermediary cluster. A third cluster (21%) was characterized by a higher frequency of compulsive buying (43%), a higher severity of compulsive buying, a stronger feeling of losing control, and higher levels of negative urgency and coping motive. These results present similarities with the Interaction of Person-Affect-Cognition-Execution (I-PACE) model of addiction and the negative reinforcement model of drug addiction, which both postulate that negative feelings play a central role in motivating and maintaining addiction. These results also echo other typologies performed in problem gamblers and problematic videogame users. These similarities of psychological profiles with other addictive behaviors, and with common symptoms and clinical expressions, are supplementary arguments to consider conceptualizing compulsive buying as an addictive disorder.

## Introduction

Compulsive buying (CB) refers to a problematic buying behavior involving frequent and excessive buying episodes, which are uncontrollable and persistent despite negative consequences. Such a disorder may induce marked clinical distress and cause significant psychological, interpersonal, and financial difficulties (1–3). In a meta-analysis, the prevalence of CB was estimated at 4.9% in a representative adult sample, with female and young individuals being more prone to develop CB (4). This disorder has been the subject of increasing attention but is under-studied and neglected in clinical settings (5). As a consequence, its classification as an individualized mental disorder, either as an impulse-control disorder, an obsessive-compulsive disorder, or an addictive disorder, has been and remains a subject of debate (2, 5–8).

Several psychological factors may be involved in the etiology of CB, such as impulsivity, material values, depression, perceived stress, poor self-esteem, decision-making difficulties, cue-reactivity reward and punishment sensitivity, distortions in judging elapsed time, *etc.* (5, 9–13). Among them, impulsivity was certainly the most studied because it may relate to the loss of control experienced by compulsive buyers (9, 14–16). As defined in the UPPS model (17), impulsivity is a multidimensional construct composed of five facets: positive and negative urgency refer to the tendency to act rashly when managing either positive or negative emotions; lack of premeditation is the inclination to act without thinking of the consequences; lack of perseverance is the inability to remain focused on a boring and/or difficult task; and sensation seeking is the tendency to enjoy and pursue new and exciting activities. Impulsivity has been consistently found to be a predictor of CB (9, 15, 16), especially the “urgency” (*i.e.*, positive and negative) dimensions. A high level of impulsivity, and especially a high urgency, may lead to poor self-control capacities and thus to an unregulated behavior (9).

According to several authors, low self-esteem is associated with CB (18–20). In a study on 548 female consumers, self-esteem was identified as a moderating factor both of the direct relationship between perceived stress and online CB and of the mediation effect of negative coping in the relationship between perceived stress and online CB (13). More specifically, Biolcati highlighted that low self-esteem is a strong predictor of CB, and that this relationship is partially mediated by the fear of others' negative judgment in women (20). As hypothesized by this author, individuals who base their self-worth on external standards are more prone to compare themselves with others and to seek their approval, leading to poor self-evaluation. Such self-disapproval may induce the process of symbolic self-completion, which implies that the acquisition of material symbols is thought to compensate for perceived inadequacies in self-evaluation (21). Such a process may lead individuals to engage in frequent and excessive purchases as an alternative solution for low self-esteem (20) and to sometimes develop CB.

Finally, motives for buying have been poorly explored in the CB literature. However, a key study based on ecological momentary assessment has demonstrated that changes in mood states were significantly associated with CB episodes (22). In particular, negative mood increased prior to and decreased following a CB episode, whereas positive mood decreased prior to a CB episode and subsequently remained stable. Moreover, the “negative affect” personality trait was significantly associated with CB in an online survey on 233 students, supporting the important of negative emotions in this disorder (23). These results may support the assumption that CB occurs mainly to obtain relief from negative feelings, as hypothesized by Billieux et al. (9). In consumer research, most of the studies have considered utilitarian and economics motives for buying (efficiency, convenience, good value for money) or hedonist and social motives in the context of business and marketing (24–27). However, in psychological research, emotionally-driven buying motives are rarely explored. Negative coping has been previously identified as a mediating factor between perceived stress and online CB in women (13). Moreover, boredom and positive emotions were more frequently associated with engagement in buying lapses (28). The three-dimensional model of motives used in alcohol research (29) and in gambling research (30) represents a good compromise to assess such motives. Indeed, this model assumes that the consumption behavior is driven by reinforcement-related motives (negative: coping; positive: enhancement) and social-affiliation-related motives.

The objective of this study was to establish a typology of buyers grounded in psychological risk factors for CB, namely, impulsivity facets, self-esteem, and motives for buying. Attempts in the literature to identify typologies of individuals with CB have been made. Most of these attempts have targeted individuals at-risk for CB or meeting the diagnostic criteria for CB (28, 31–34), with the objective of identifying clusters of patients who can benefit from different clinical approaches. Another study has explored the heterogeneity of individuals who presented with few difficulties related to their buying behavior to discuss the relative contribution of compulsivity and impulsivity for the heterogeneity of compulsive buyers (35). Finally, another study identified three clusters of frequent buyers with moderate risk of CB or presence of CB, based on their propensity to engage in buying lapses in response to three affective states (boredom, negative emotions and positive emotions): “escape seekers”, “excitement seekers” and “low affect management buyers” (28). “Excitement seekers” were found to engage more frequently in buying lapses in response to boredom, whereas “escape seekers” engaged more frequently in buying lapses in response to negative affective states.

Given the commonalities shared between CB and addictive disorders, especially behavioral addictions (5, 36–38), a tempting approach to relate CB to addictive disorders would be based on the confirmation of common symptoms. However, such a confirmatory approach pre-assuming the addictive nature of CB may lead to biased conclusions (39). By contrast, this work proposes adopting an exploratory approach based on relevant psychological factors that have been demonstrated to be related to CB. Such a clustering approach may highlight meaningful subgroups of individuals, as has been performed for other behavioral addictions such as gambling disorder (40) and online gaming problematic involvement (41). The combination of impulsivity, buying motives and self-esteem as predictors for establishing such subgroups may highlight new insights on the profiles of buyers as compared with previous works in the field. Moreover, performing such work in a community sample with a large range of CB severity, including non at-risk buyers, may bring new insights on buying determinants, without focusing only on those who encounter problems with their buying behavior.

Our hypotheses were that we would be able to identify at least two profiles of buyers: (i) one profile with a low frequency of CB, characterized by low levels of impulsivity, medium to high self-esteem, and a buying behavior not driven by reinforcement or social affiliation (*i.e.*, low levels on the three buying motives); and (ii) one less represented profile with a higher frequency of CB and high levels of impulsivity, especially regarding urgency (*i.e.*, positive and negative) dimensions, reduced self-esteem, and high levels of motives for buying, especially reinforced motives (coping and enhancement).

## Methods

### Participants and Procedure

The participants were recruited through the internet (mainly social networks) and within the registry of volunteers for research that was constituted by our research team. For online social networks, we posted a message which presented the study, including the link to complete the survey. For the registry of volunteer, we send an email offering to participate in this survey, also including the link to complete the survey. The data collection was based on an anonymous internet survey (GoogleForms^®^). Inclusion criteria were women who aged over 18 years. No exclusion criteria were applied.

### Measures

#### Socio-Demographics

A brief questionnaire was created to assess gender, age, education level, current professional status, and level of income.

#### Buying Habits

Participants had to indicate their frequency of buying in shops and on the internet. From this data, we extracted a binary variable depending on whether the participant makes purchases once per week or more, that is, in shops or on the internet.

#### Compulsive Buying Scale (CBS)

The CBS (3) is a self-report questionnaire used to screen for CB in the general population and determine the severity of the disorder in a dimensional approach. Respondents are asked to answer a set of seven items on a 5-point Likert scale. The items explore behaviors, feelings, and motivations associated with CB. We used a French version of the CBS, composed of the 7 items proposed by Faber and O'Guinn translated into French. A bilingual English–French professional translator back translated the French items and all discrepancies between the original English CBS and the back-translation were discussed until a satisfactory solution was found.

In our sample, the CBS had a Cronbach's alpha of 0.79. According to the original version of the CBS, a total score is computed with a ponderation on each item and ranges from −7.03 to +3.61. Participants who scored lower than −1.34 were classified as individuals with CB (CB group), and participants who scored higher were classified as individuals without CB (non-CB group). In addition to the CBS, participants were asked to estimate their feeling of losing control over buying behavior in the 6 previous months on a Visual Analogic Scale from 0 (*no loss of control*) to 10 (*complete loss of control*).

#### Buying Motives Questionnaire (BMQ)

The BMQ is a direct adaptation from the Gambling Motives Questionnaire (30), with instructions adapted to buying behaviors. The formulation of items in French were obtained from the French version of the Gambling Motives Questionnaire (42). This 15-item self-report questionnaire is for the assessment of motives for buying according to three dimensions: coping (buying to relieve negative feelings or boredom, or to escape real-life problems), which is a negative reinforcement-related motive; enhancement (buying to search for stimulation, arousal, or positive feelings), which is a positive reinforcement-related motive; social (buying to enhance affiliation with others or to share moments with peers). Participants are asked to indicate how often they buy for each of the 15 listed reasons with a 4-point Likert scale. The score of each dimension ranges from 5 to 20. In our sample, reliability of the BMQ was found to be good, with a Cronbach's alpha of 0.88. The BMQ subscales displayed moderate (social motive, alpha = 0.49) to good reliability (coping motive, alpha = 0.85; enhancement motive, alpha = 0.82).

#### Short UPPS-P Impulsivity Scale (UPPS-P)

We used the French version of the UPPS-P (43), adapted from the original English version (44). It is a 20-item self-report questionnaire used to measure five dimensions of the UPPS model of impulsivity (17): positive and negative urgency, (lack of) premeditation, (lack of) perseverance, and sensation seeking. Participants were asked to answer on a 4-point Likert scale, and a score ranging from 4 to 16 was computed for each dimension. The UPPS-P had good reliability in our sample (positive urgency, alpha = 0.84; negative urgency, alpha = 0.76; lack of premeditation, alpha = 0.83; lack of perseverance, alpha = 0.90; sensation seeking, alpha = 0.86).

#### Rosenberg Self-Esteem Scale (RSES)

The RSES (45) is a 10-item self-report scale, which assesses the global self-esteem on a 4-point Likert scale. The total score is computed by summing the scores of all the items, with half of them being reversed. A score lower than 30 indicates low self-esteem. A validated French version of this scale was used for our study (46) and had excellent reliability (Cronbach's alpha of 0.91).

### Statistical Analysis

A descriptive analysis of the sample was first conducted.

A typology of participants was then determined using Latent Class Cluster Analysis (LCCA) (47). The purpose of using LCCA was to classify similar observations into homogeneous groups, where the number of groups is unknown. Unlike other clustering algorithms such as K-means or hierarchical clustering, a probabilistic model is used to attribute to each observation a set of cluster membership probabilities, rather than affecting each individual to a unique cluster. Consequently, each individual is characterized by as many membership probabilities as there are clusters. For example, if the final model is composed of three clusters (A, B, and C), a participant will be characterized by three probabilities of being in each cluster (for example, P_A_ = 40%; P_B_ = 15%; P_C_ = 45%). This strategy is particularly notable for avoiding a loss of information. Indeed, in the provided example, the participant would have been classified in cluster C with traditional clustering algorithms, while his probability of being in cluster A was almost equivalent.

The variables included in the LCCA were the Z-transformed scores of the psychological factors: the RSES score, the three BMQ scores, and the five UPPS-P scores. This strategy was used to avoid the problems related to the different ranges of the three scales and to allow for the observation of the relative contribution of each score to the clustering. The Z-transformation was applied with reference to means and standard deviations of the non-CB group; thus, the “standard” adopted is that of individuals with non-CB behavior. We fitted 1- to 7- cluster models using maximum *a posteriori* estimation and selected the best model as the one with the lowest Bayesian information criterion (BIC). Other fit indices were also computed to reflect the accuracy of classification, namely entropy (which should be the closest to one as possible) and classification error rate (which should be the closest to zero as possible).

The clusters were compared using one-factor analyses of variance (ANOVAs) on the psychological factors used for the clustering (impulsivity, motives, and self-esteem) and the covariates of interest, that is, age, frequency of buying, and severity of CB (CBS score, CBS category, and loss of control score). The variables used for the ANOVAs were the weighted scores according to the cluster membership probabilities in each cluster (*i.e*. in each cluster, the score of a participant was re-estimated using the membership probability of the participant in the specified cluster). In case of significance, comparisons between all possible pairs of means of the three clusters were conducted using *post-hoc* Tukey (HSD) tests.

### Ethics

The study was conducted in accordance with Good Clinical Practice Guidelines and the Declaration of Helsinki, with approval from the local ethics committee (Groupe Nantais d'Ethique dans le Domaine de la Santé—GNEDS, Nantes). All participants provided electronic informed consent (individuals who were minor or under guardianship were not included). No compensation was given for participation.

## Results

### Description of the Sample

A description of the total sample is provided in **Table 1**. Among the 242 participants, 34 were identified as individuals with CB and 208 as individuals without CB. Due to the recruitment through social networks, most participants were students aged around 25 years old, with a low level of income.

**Table 1:** Description of the sample (n = 242).

	M (SD) or N (%)
Age	25.6 (9.6)
Education level (years)	14.7 (1.7)
Professional status	
Student	176 (72.4%)
Active	56 (23.0%)
Inactive	8 (3.3%)
Retired	2 (0.8%)
Income	
≤€540^a^	142 (58.7%)
≤€1150^b^	48 (19.8%)
≤€2300	39 (16.1%)
>€2300	13 (5.4%)
CBS status	
Non-CB	208 (86.0%)
CB	34 (14.0%)

^a^In France, a €540-income corresponds to the RSA (Active Solidarity Income), which is provided to individuals without financial resources to guarantee them a minimum level of income; ^b^In France, a €1150-income corresponds to the SMIC (Growth-Indexed Minimum Wage), which is the minimum wage that a person with an employed activity must perceive as a salary.

### Typology of Buyers: Results of the LCAA

The fit indices of the 1- to 7- cluster solutions tested in the Latent Class Cluster Analysis (LCCA) are provided in **Table 2**. The three-cluster solution was selected because it had the lowest BIC. The entropy of the selected model was 0.801, which is considered high entropy (48), and the classification error rate was low (8.7%). This means that the latent classes were well distinct and that class membership probabilities were well predicted.

**Table 2:** Fit indices for 1- to 7- cluster solutions.

	Log-likelihood	BIC	Entropy	Classification errors
**1-Cluster**	−4691,17	9470,17	1	0
**2-Cluster**	−4510,12	9201,37	0.802	0.056
**3-Cluster**	−4439,57	9153,58	0.801	0.087
**4-Cluster**	−4403,24	9174,24	0.812	0.097
**5-Cluster**	−4352,38	9165,82	0.831	0.096
**6-Cluster**	−4345,78	9245,95	0.828	0.112
**7-Cluster**	−4327,15	9302,00	0.865	0.093

BIC, Bayesian information criterion.

**Figure 1:**
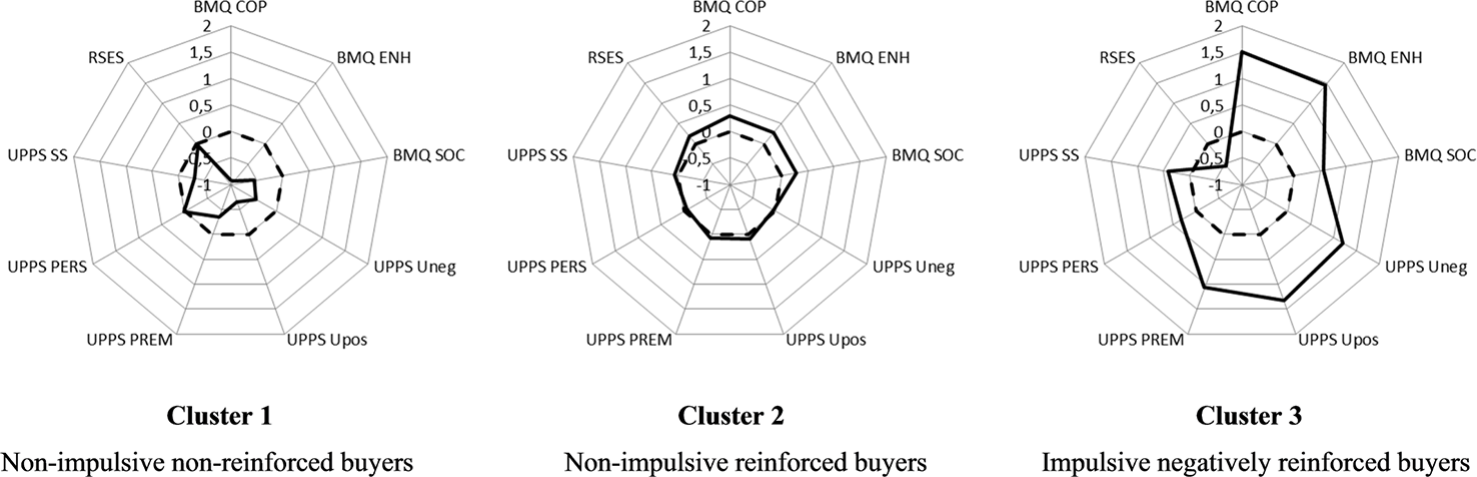
Profiles of the three clusters of buyers. UPPS, Short UPPS-P Impulsivity Scale; BMQ, Buying Motives Questionnaire; RSES, Rosenberg Self-Esteem Scale. BMQ COP, Coping score of the BMQ; BMQ ENH, Enhancement score of the BMQ; BMQ SOC, Social score of the BMQ; UPPS Uneg, Negative Urgency score of the UPPS; UPPS Upos, Positive Urgency score of the UPPS; UPPS PREM, Lack of Premeditation score of the UPPS; UPPS PERS, Lack of Perseverance score of the UPPS; UPPS SS, Sensation Seeking score of the UPPS; RSES = total score of the RSES. Dashed lines represent the mean Z-scores of the non-CB group, which was used as the standard. Bold lines correspond to mean Z-scores of each cluster.

The profiles of the three clusters obtained with the LCCA are depicted in **Figure 1**. Compared with the non-CB group (dashed line), individuals from Cluster 1 seem to display lower scores on all the variables, except self-esteem and perseverance. However, the Z-scores are all comprised between 0 and −1 standard deviation (SD), so that the observed differences are not considered clinically meaningful. Individuals from Cluster 2 seem to have a profile very similar to that of the non-CB group, with SD very close to zero. Finally, individuals from Cluster 3 seem to have a very different profile than the non-CB group, *i.e.* largely higher scores for buying motives and impulsivity, and lower self-esteem scores. The Z-scores are comprised between +1 and +1.5 SD for coping and enhancement motives, and for positive urgency, negative urgency, and lack of premeditation, which is considered close to clinical meaningfulness. However, as the relative proportion of non-CB individuals is very different from a cluster to another (see below), these results should be interpreted with caution.

The results from the ANOVAs are presented in **Table 3**. Among the psychological factors that contribute to the clustering, the three clusters differed significantly regarding three impulsivity facets (negative urgency, positive urgency, and lack of premeditation) and two buying motives (coping and enhancement) but not self-esteem. We observed a gradient from Cluster 1 to Cluster 3 (Cluster 1 < Cluster 2 < Cluster 3) for all the scores with significant differences.

**Table 3:** Description and comparisons of the three clusters of buyers.

Weighted scores^a^	Cluster 1 Nonimpulsive nonreinforced buyers	Cluster 2 Nonimpulsive reinforced buyers	Cluster 3 Impulsive reinforced buyers	ANOVAs		*post-hoc* Tukey (HSD) tests
Average cluster size^b^	0.28	0.51	0.21		
**Psychological factors** (contributive to the clustering)	M (*SD*)	M (*SD*)	M (*SD*)	F	p values	p values ^c^
Impulsivity						
UPPS—negative urgency (/16)	7.84 (*12.05*)	8.97 (*7.90*)	12.36 (*22.99*)	5.47	**0.004**	1–2: 0.709 **1**–**3: 0.004** **2**–**3: 0.045**
UPPS—positive urgency (/16)	8.79 (*13.11*)	10.52 (*9.15*)	13.40 (*24.32*)	4.66	**0.010**	1–2: 0.491 **1**–**3: 0.007** 2–3: 0.143
UPPS—lack of premeditation (/16)	6.53 (*10.01*)	7.39 (*6.65*)	9.44 (*18.22*)	3.41	**0.034**	1–2: 0.738 **1**–**3: 0.030** 2–3: 0.171
UPPS—lack of perseverance (/16)	7.20 (*11.66*)	6.99 (*6.46*)	7.95 (*15.70*)	0.44	0.644	–
UPPS—sensation seeking (/16)	9.16 (*14.07*)	10.20 (*9.27*)	11.22 (*21.20*)	1.05	0.349	–
Buying motives						
BMQ—coping (/20)	6.38 (*9.32*)	10.30 (*9.60*)	14.18 (*26.39*)	12.60	**< 0.001**	**1**–**2: 0.031** **1**–**3: < 0.001** **2**–**3: 0.033**
BMQ—enhancement (/20)	6.36 (*9.32*)	9.85 (*9.04*)	13.27 (*24.97*)	10.93	**< 0.001**	**1**–**2: 0.048** **1**–**3: < 0.001** 2–3: 0.054
BMQ—social (/20)	7.47 (*11.16*)	9.13 (*8.25*)	9.69 (*17.84*)	1.90	0.150	–
Self-esteem						
RSES—total score (/40)	28.76 (*44.48*)	30.21 (*26.97*)	25.56 (*46.20*)	0.85	0.429	–
**Covariates** (not contributive to the clustering)						
Age	27.70 (*46.19*)	25.39 (*25.18*)	23.58 (*43.44*)	0.66	0.515	–
Frequency of buying (once per week or more)	0.04 (*0.39*)	0.05 (*0.31*)	0.20 (*0.92*)	5.01	**0.007**	1–2: 0.980 **1**–**3: 0.022** **2**–**3: 0.013**
Severity of CB						
CBS score	2.03 (*3.88*)	1.07 (*2.10*)	-1.12 (*4.70*)	45.66	**< 0.001**	**1**–**2: 0.012** **1**–**3: < 0.001** **2**–**3: < 0.001**
CBS category (probability to be an individual with CB)	0.02 (*0.23*)	0.08 (*0.36*)	0.43 (*1.32*)	18.68	**< 0.001**	1–2: 0.670 **1**–**3: < 0.001** **2**–**3: < 0.001**
Loss of control score (/10)	1.32 (*4.08*)	2.30 (*3.43*)	4.08 (*9.66*)	11.65	**< 0.001**	1–2: 0.208 **1**–**3: 0.006** **2**–**3: < 0.001**

UPPS, Short UPPS-P Impulsivity Scale; BMQ, Buying Motives Questionnaire; RSES, Rosenberg Self-Esteem Scale; CBS, Compulsive Buying Scale; CB, Compulsive Buying; ^a^The weighted scores for a given cluster were computed by multiplying the raw score of each individual by the membership probability in the given cluster and by dividing the result by the mean of the membership probabilities of the given cluster of all the individuals. Given the membership probability weight, the distribution of weighted scores is artificially more dispersed; ^b^The average cluster size represents the mean of the cluster membership probabilities of all the participants for each cluster; ^c^For each variable, p values are reported for the comparisons of all possible pairs of means of the three clusters (1–2: cluster 1 vs cluster 2; 1–3: cluster 1 vs cluster 3; 2–3: cluster 2 vs cluster 3); Significant p values (p < 0.05) are indicated in bold.

More specifically, *post-hoc* analyses revealed that individuals from the Cluster 3 scored significantly higher than the two other clusters on the UPPS negative urgency dimension and on the BMQ coping dimension. The other observed differences (positive urgency, lack of premeditation, and enhancement motive) were only in comparison with Cluster 1, which is the lowest extremity of the gradient. Consequently, Cluster 3 was named “impulsive reinforced buyers.” Moreover, this cluster was the cluster that displayed the highest probability of buying once per week or more; the lowest CBS score, that is, the higher the severity of CB, the higher probability of being an individual with CB, and the highest feeling of losing of control.

The only characteristics which distinguished Cluster 2 from Cluster 1 in the *post-hoc* analyses were the BMQ coping and enhancement dimensions, with Cluster 2 scoring higher for both. This cluster was therefore named “non-impulsive reinforced buyers.”

Finally, Cluster 1 was characterized by low levels of impulsivity and buying motives; thus, this cluster was named them “nonimpulsive nonreinforced buyers.”

When considering only individuals with CB (*n* = 34), we found an increasing probability in each cluster, with a gradient from Cluster 1 to Cluster 3: Cluster 1 (4.1%) < Cluster 2 (30.2%) < Cluster 3 (65.7%).

## Discussion

The objective of the present work was to investigate the profiles of buyers according to psychological characteristics known to be associated with a higher risk of CB (buying motives, impulsivity, and self-esteem). We identified three clusters of buyers with an observed gradient of impulsivity (negative urgency, positive urgency, and lack of premeditation) and buying motives (coping and enhancement).

As expected, we were able to isolate a non-problematic cluster, the “non-impulsive non-reinforced buyers” cluster, which represented a quarter of the sample. This cluster was characterized by a very low frequency of CB (2%), low levels of impulsivity, medium self-esteem (weighted score just under the threshold for “high self-esteem”), and low levels of buying motives for the three dimensions. Given the very low buying motives scores, an assumption could be that those individuals make purchases for a primarily utilitarian purpose and do not expect any rewards from the buying behavior other than the good.

In addition to this non-problematic cluster, an intermediary cluster emerged: the “non-impulsive reinforced buyers” cluster. This cluster was also characterized by a low frequency of CB (8%), with low to medium levels of impulsivity and high self-esteem (weighted score just over the threshold). Compared with the first cluster, “non-impulsive reinforced buyers” displayed higher levels of reinforcement-related buying motives, that is, negatively reinforced (coping) and positively reinforced (enhancement) motives, and a higher severity of CB (although insufficient to reach the threshold for CB). This cluster was the most represented among the sample (51%) and may represent a category of buyers experiencing few difficulties with their buying behavior (higher severity of CB than the first cluster) and with no real clinical distress. For these individuals, buying episodes may have either positive or negative reinforcing properties.

Regarding the literature on CB, such emotionally-driven buying behaviors can induce a decrease in self-regulation capacities (22), especially for negative mood. As a consequence, and given the higher severity of CB evidenced in this cluster compared with the nonproblematic cluster, “nonimpulsive reinforced buyers” may be more at-risk for subsequent development of CB. However, it is critical to highlight that those buyers are primarily non-problematic buyers with a weak sense of losing control over the buying behavior, similar to the first cluster, and a low frequency of CB (8%). As a consequence, no real psychological distress can be evidenced.

Finally, as expected, we were able to identify a cluster with a high frequency of CB (43%), which was characterized by higher impulsivity, stronger buying motives, low self-esteem, an elevated feeling of losing control over the buying behavior, and a higher level of CB severity. Although not directly assessed, this may presume psychological distress for a non-negligible proportion of buyers in this cluster, and those buyers may be identified as “problematic” buyers. Although being the least represented cluster, “impulsive reinforced buyers” nevertheless represented approximately one fifth of the sample (21%), which was much more than expected. Buyers from this cluster were especially distinguished from the intermediary cluster regarding negative urgency and coping motive. They displayed a much higher negative urgency score, which is relevant to the assumption made by Billieux et al.: buyers with elevated negative urgency are more likely to buy compulsively when they experience negative feelings (9).

Moreover, this higher responsiveness to negative feelings is supported because those buyers who have the highest levels of impulsivity also displayed the highest levels of coping motives. Indeed, in the literature regarding CB, buying motives have been little explored, especially with the three-dimensional model of motives used in the addictive literature (29, 30). More specifically, the coping motive refers to the completion of the behavior to relieve negative feelings or boredom, or to escape real-life problems. “Impulsive reinforced buyers” may thus use buying as a maladaptive coping strategy to manage negative feelings, as demonstrated by Muller et al., who used ecological momentary assessment (22).

Such a switch to negatively reinforced behaviors for problematic subgroups has been previously highlighted in problem gamblers (49) and problematic videogame users (41), which would put forward a certain form of similarity with behavioral addictions. This result is also in line with the recently revised I-PACE model of addiction (50). Such model postulates that the addictive behavior results from the interaction of a person's core characteristics that predispose to addiction (such as genetics, psychopathology, temperament, coping style, motives for using, *etc.*), affective and cognitive responses to external or internal triggers, and executive functioning (inhibitory control and decision-making). According to this model, the addictive behavior is learnt in early stages through changes of expectancies (feelings of gratification or relief from negative moods in response to the behavior) in response to internal and external triggers associated with the behavior. Such changes may increase the urge to perform the behavior in subsequent confrontation to the triggers. In later stages, gratification gradually switches to compensation, and stimuli-specific inhibitory control is altered, leading to a progressive loss of control over the urges and the maintenance of the behavior. As a consequence, in such later stages, negative moods and dysfunctional coping skills play a central role in maintaining addictive behaviors (50, 51). Moreover, as hypothesized by Baker et al. in the framework of the negative reinforcement model of drug addiction, the induction of negative feelings through exposure to negative cues should increase motivation for buying (52). In our study, the cluster with the higher frequency of CB is the cluster with the higher levels of negative buying motives and negative urgency, which seems compatible with the central role of negative reinforcement highlighted in addictive disorders. Moreover, still according to Baker et al., in the context of drug addiction, abstinence from buying should induce a progressive increase in negative feelings and attention to negative cues, either internal or external cues (52). Such assumptions should be more deeply explored and confirmed in future studies on CB, especially with neurocognitive approaches and clinical samples. However, the similarities of the psychological profiles of individuals with CB with other addictive behaviors, and common symptoms and clinical expressions, are supplementary arguments to consider conceptualizing CB as an addictive disorder, as argued by several researchers (5, 7).

This work has several limitations. First, the sample was recruited from two sources (online networks and registry of volunteer). This may have brought a kind of heterogeneity in the profiles of participants. Second, the majority of the sample was students, and the sample was a self-selected, which may limit the generalizability of the results. Third, considering that the data collection was based on a cross-sectional design and included only self-reported questionnaires, the results should be considered as preliminary and considered with caution. Moreover, the survey was limited to only measures necessary to explore our hypotheses. However, nor psychiatric symptoms such as depression or anxiety nor co-addictions were screened (for the sake of feasibility of the survey, which had to remain short). Future studies interested in subtyping CBr should use longitudinal designs to explore the relative role of buying motives, especially negative buying motives; impulsivity; and self-esteem on the subsequent development of CB, taking into account psychiatric or addictive comorbidities. Fourth, the sample was composed of only women, which may have reduced the scope of the results. However, CB is a disorder with a large predominance of women. Fifth, the BMQ explores only positive and negative reinforcement-related and social-affiliation-related motives. In the same manner as what was performed for the Gambling Motives Questionnaire with the addition of a financial motive (53), it may be useful to develop a revised version of the BMQ which explores other motives relevant for buying behavior, and especially utilitarian motives, which are a critical motivation for everyday purchases. Moreover, as the BMQ relies on a model developed for substance use, one could argue that its use may participate in a confirmatory approach that we tried to avoid. However, the BMQ model explores motives for use (consumption), and not especially for pathological/addictive use. As a consequence, it does not explore symptoms of CB in an addictive perspective, but rather the reasons to engage in buying. It was especially relevant because it gave us access to emotionally-driven (positive and negative) motives. Finally, this work was performed on a community sample. We cannot exclude that the profile obtained will not be similar to a clinical sample. Moreover, the sample size of the sample was marginally sufficient in size for this type of analyses. As a consequence, the present sample should not be considered representative of the population of compulsive buyers. However, the objective here was to highlight profiles of buyers according to certain vulnerability traits and to observe whether such profiles show different distributions of CB.

## Conclusion

Our study has allowed the identification of three profiles of buyers, who differed significantly in impulsivity and buying motives. A problematic cluster emerged, characterized by a high frequency of CB and an association with buying motives and impulsivity with negative feelings. These results present similarities with certain aspects of the I-PACE model of addiction and echo other typologies performed with problem gamblers and problematic videogame users. These similarities of psychological profiles with other addictive behaviors, and with common symptoms and clinical expressions, are supplementary arguments to consider conceptualizing CB as an addictive disorder.

## Data Availability Statement

The raw data supporting the conclusions of this article will be made available by the authors to any qualified researchers, provided that the intended purpose of use is relevant with the ethical consent given by the participant and with French legislation. Requests to access the dataset analyzed in this study should be directed to the corresponding author.

## Ethics Statement

The studies involving human participants were reviewed and approved by Groupe Nantais d'Ethique dans le Domaine de la Santé—GNEDS, Nantes. The patients/participants provided their written informed consent to participate in this study.

## Author Contributions

GC-B, JM, and EC designed the study. GC-B was responsible for the project management and supervision. JM created the Internet survey, conducted the data collection, and performed the descriptive analysis. BP conducted the Latent Class Analysis. EC and GC-B were responsible of the interpretation of data and wrote the first draft of the manuscript. MG-B provided significant feedback on the manuscript. All authors had full access to all data and approved the final manuscript.

## Conflict of Interest

The authors declare that the research was conducted in the absence of any commercial or financial relationships that could be construed as a potential conflict of interest.
